# Investigating the causal relationship between physical activity and incident knee osteoarthritis: a two-sample Mendelian randomization study

**DOI:** 10.1038/s41598-024-52175-4

**Published:** 2024-01-18

**Authors:** Liufang Huang, Yuling Zhang, Qian Li

**Affiliations:** Department of Rehabilitation Medicine, People’s Hospital of Guanghan City, 75 Hankou Road, Luocheng Town, Guanghan City, Sichuan Province People’s Republic of China

**Keywords:** Diseases, Risk factors

## Abstract

There is evidence that physical activity (PA) has a long-term positive impact on disease. Whether PA is a risk factor for knee osteoarthritis (OA) is still controversial. The purpose of this study was to explore whether there is a causal relationship between PA and knee OA. We extracted PA and knee OA data from genome-wide association study (GWAS) databases. We used single-nucleotide polymorphisms (SNPs) as instrumental variables. We performed MR analysis by random-effects inverse-variance weighting (IVW), MR‒Egger, weighted median, simple mode, and weighted mode methods. We evaluated the stability and reliability of the results through sensitivity analysis. There was no significant association between PA and knee OA (p > 0.05). We did not detect any pleiotropy (MR‒Egger intercept test et al.: p > 0.05). The sensitivity analysis confirmed our results (p > 0.05). There is no causal relationship between PA and knee OA.

## Introduction

Osteoarthritis (OA), a common disease, not only causes pain in the joints but can also lead to a decrease in joint function, and further progression can cause disability^1^. Due to factors such as obesity and aging, the number of people suffering from OA is expected to rise in the future, and OA will afflict an increasing number of patients. OA not only causes physical pain to the patients themselves but also imposes a heavy financial burden on their families, with a noticeable impact in terms of socioeconomic costs and the health care system^2,3^. OA treatment methods include physical therapy, drug therapy, surgery and so on. However, these treatment methods only relieve pain symptoms and ultimately increase the economic burden of patients^4^. Therefore, Therefore, the pathogenesis and pathogenic factors of OA are receiving increasing attention to reduce its occurrence by controlling risk factors.

OA most commonly occurs in the knee. Previous knee injury, female sex, and obesity have been identified as potential risk factors for the development of knee OA^5,6^. Research has shown that physical activity (PA) has a long-term positive impact on diseases such as coronary heart disease^7^. PA also plays a role in the mental state of elderly people^8^. Despite the benefits associated with PA, whether it is a risk factor for OA (especially knee OA) is still highly controversial^9,10^. In addition, physiotherapists promote aerobic exercise in the treatment of OA^11^. Therefore, it is important to determine whether there is a causal relationship between PA and knee OA.

Currently, Mendelian randomization (MR) has been used extensively in genetic epidemiology research to explore risk factors associated with disease^12^. MR includes the use of genetic variants [instrumental variables (IVs)] to determine causality in the relationships between exposures and outcomes. The main advantage of this approach is that it avoids the creation of bias and the influence of potential confounding factors that are found in traditional research methods. Also, reverse causation does not typically occur in MR studies^12,13^.

Since it is still unclear whether PA is a risk factor for knee OA, there is a lingering concern that PA contributes to the development of knee OA. Therefore, we used two-sample MR analysis to explore whether there is a causal relationship between PA and knee OA.

## Methods

### Data availability

The data we used for this study were all obtained from published studies that were accessed from publicly available databases (most recent data publicly available in public databases). UK Biobank received ethical approval from the Research Ethics Committee, and all participants provided written, informed consent. The original studies also had ethical approval from the relevant institutions and consent from the participants themselves. This study was conducted in accordance with Burgess's guidelines and was reported in accordance with the STROBE-MR statement.

### Exposure data

Single-nucleotide polymorphisms (SNPs) for this study were obtained from accessible and publicly available genome-wide association studies (GWAS) related to knee OA. Valid IVs should fulfill three assumptions: the IVs are strongly correlated with exposure; the IVs are not interfered with by any confounding factors; and the IVs affect the outcome only through exposure. We selected SNPs that were closely associated with exposure at the genome-wide significance level under stringent conditions (p < 5 × 10^–8^). For the independence of SNPs, we used the following conditions: clumping window, 10,000 kb; r^2^ 0.001. The data for PA were derived from Wang et al.'s summary of relevant data from databases such as UK Biobank (https://www.ebi.ac.uk/gwas/publications/36071172)^14^. The primary study population for this GWAS comprised people of European ancestry. The number of PA patients was 606,820, and the number of non-PA patients was 526,725. The intensity of PA was self-reported as moderate-to-vigorous PA. In addition, to avoid the influence of SNPs related to aggravating existing/existing OA, we identified and excluded relevant SNPs by searching related articles published in PubMed.

### Outcome data

The data for knee OA were derived from Zengini et al.'s summary of relevant data from databases such as UK Biobank (https://www.ebi.ac.uk/gwas/publications/29559693)^15^. Similarly, the main subjects of their research are people of European ancestry. There were 4672 knee OA patients and 172,791 individuals in the control group.

Although we used exposure and outcome data from papers published in UK Biobank, the original samples for both exposure and outcome data were all from different research institutions, and the original samples did not overlap. Therefore, there are no duplicate samples in the exposure and outcome data.

### Statistical analysis

We analyzed the collected data with the “TwoSampleMR” package in R software (version 4.3.1). For exposed IVs, we selected SNPs with genome-wide significance (p < 5 × 10^–8^; r^2^ = 0.001, kb = 10,000)^16^. We then extracted IV-related data (without the use of proxy SNPs) from the knee OA outcome dataset. We harmonized exposure and outcome datasets. To determine causality in the association between PA and knee OA, we mainly used the random-effects inverse-variance weighting (IVW) model (for the IVW method, its accuracy and stability were based on the fact that all IVs are valid and there is no directional pleiotropy). MR‒Egger, weighted median, simple mode, and weighted mode were used to supplement our analysis. The primary method for detecting directional horizontal pleiotropy was the MR Egger intercept test (an intercept that was not equal to 0 was considered to be free of directional horizontal pleiotropy). In addition, the symmetry of the funnel plot was used to assess directional pleiotropy. For the assessment of pleiotropy, we also used the leave-one-out sensitivity test as well as the MR-PRESSO test. The heterogeneity of individual effects for each gene variant was assessed by using Cochran's Q statistic. A statistically significant difference was indicated by p < 0.05. The results were expressed as odds ratios (ORs) and corresponding 95% confidence intervals (Cis).

## Results

### MR analysis

Through rigorous screening, 17 SNPs with strong correlations were finally used as instrumental variables between PA and knee OA (F-statistic > 10). We did not find a statistically significant association between PA and knee OA by using the IVW model method (p = 0.918). Similarly, the MR‒Egger, weighted median, simple mode, and weighted mode methods used in this study revealed a statistically significant association between PA and knee OA (p > 0.05). Table 1 shows the results of the two-sample MR analysis between PA and knee OA. The included SNPs are shown in Supplementary Table 1.

**Table 1:** The results of the two-sample mendelian randomization analysis.

Exposures	Outcomes	IVW	Weighted median	MR Egger	Weighted mode	Simple mode
		P	P	P	P	P
PA	Knee OA	0.918	0.409	0.527	0.580	0.635

*PA* physical activity, *knee OA* knee osteoarthritis, *IVW* random-effect inverse variance weighted.

### Sensitivity analysis

**Figure 1:**
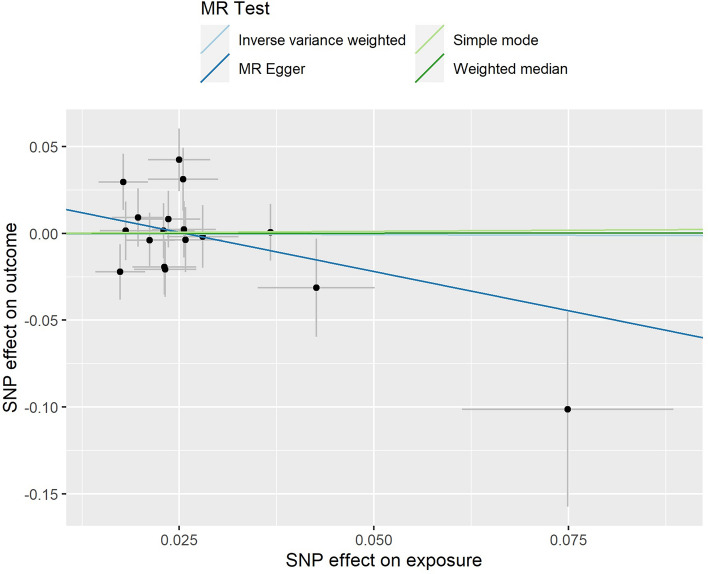
The results of MR‒Egger regression. *MR* Mendelian randomization.

**Figure 2:**
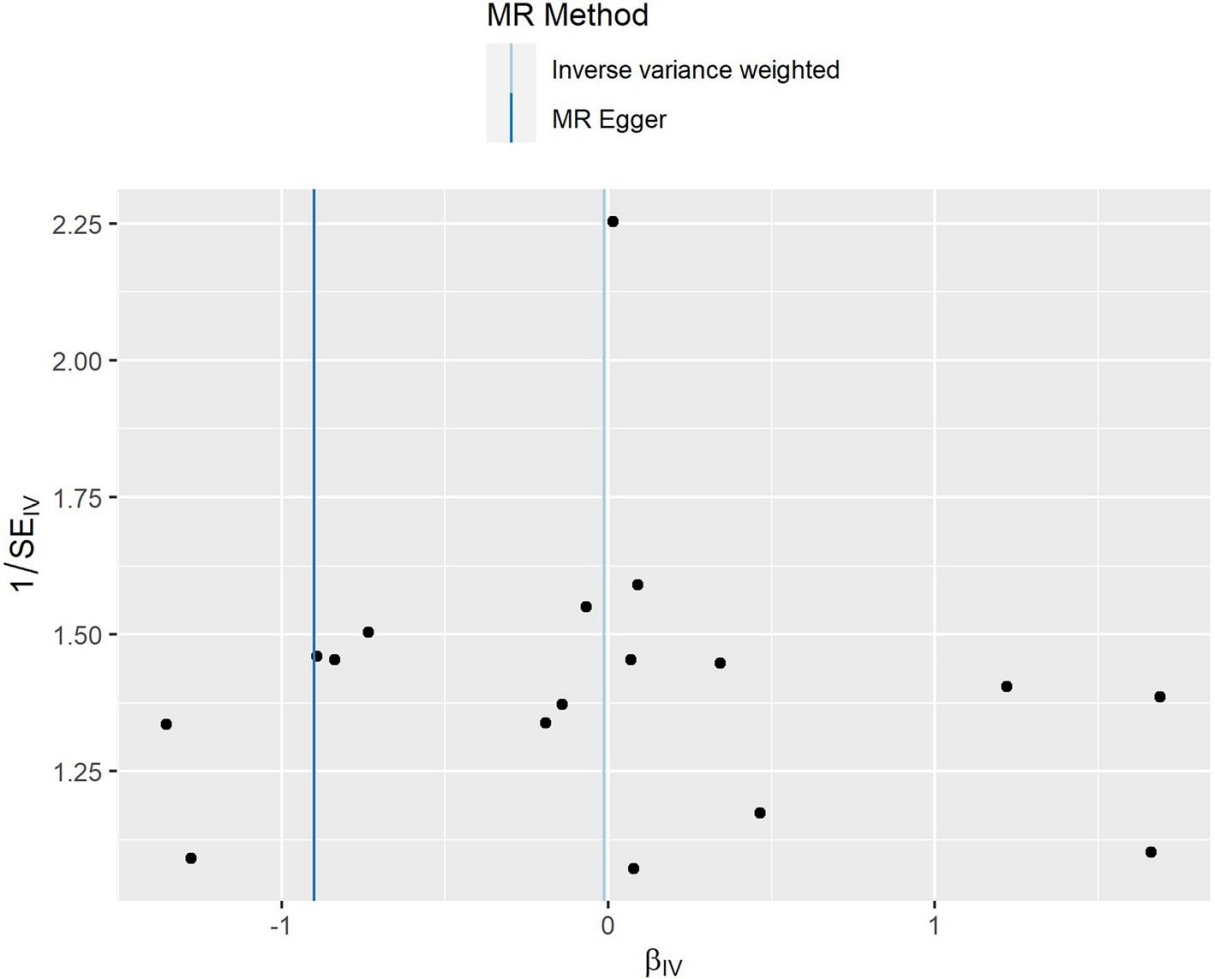
The results of funnel plot. *MR* Mendelian randomization.

**Figure 3:**
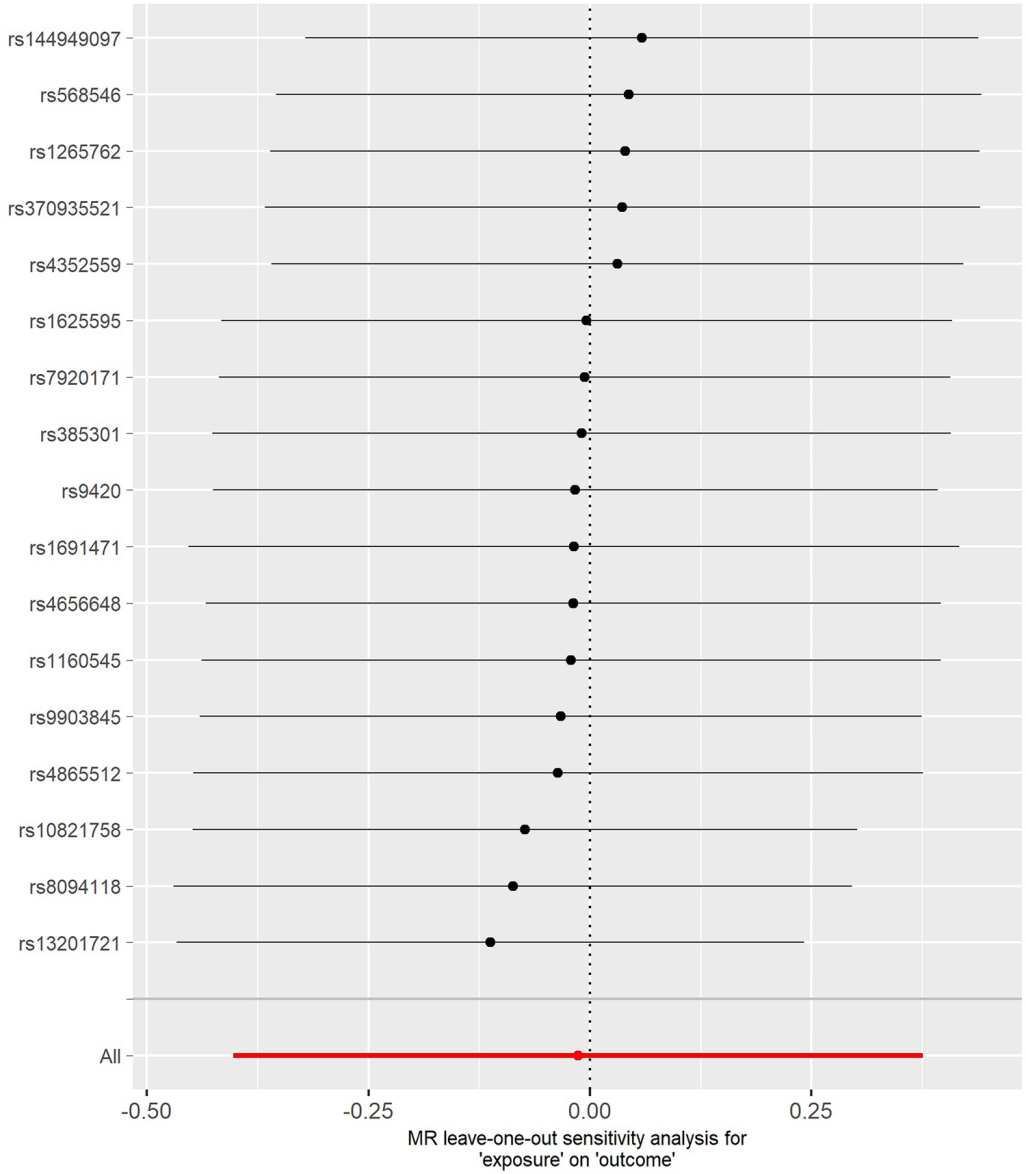
MR leave-one-out sensitivity analysis. *MR* Mendelian randomization.

The results of the MR‒Egger intercept test suggested that no pleiotropy occurred (p > 0.05) (Fig. 1). Similarly, the results of the funnel plot suggested a very low risk of pleiotropy (Fig. 2). The results of the leave-one-out sensitivity tests and MR-PRESSO also revealed no pleiotropy (Fig. 3). There was also no statistical significance of the Cochran's Q statistic for heterogeneity.

## Discussion

We used GWAS data from a large sample to explore the causal relationship between PA and knee OA. The results of this study suggest that there is no significant causal relationship between PA and knee OA. In other words, PA did not increase the risk of developing knee OA. In summary, PA was not a risk factor for the development of knee OA. Our results were also relatively stable in the sensitivity analysis.

There has been a well-known controversy over whether there is a causal relationship between PA and knee OA^9,10^. However, there were differences in the definition and degree of PA in different studies. In addition, there were differences in the methods used to study knee OA^17^. In addition, McAlindon et al.^18^ found an association between high-intensity PA and the risk of developing knee OA. While that association is possible, notably, the PA duration was a part of the equation that cannot be ignored. Some studies have shown that prolonged PA time will increase the probability of knee OA^19,20^. In addition, some of the current studies have included some self-reported data^20^. Self-reporting can also lead to differences in results due to the presence of subjective factors. For different populations, different occupations are also an important factor in the prevalence of knee OA. Manual workers have a higher risk of developing knee OA than nonmanual workers^21^.

The current research primarily suggests that PA will not increase the incidence rate of knee OA. The meta-analysis by Coburn et al. and Gates et al. yielded the same result: PA is not a risk factor for the development of knee OA^22,23^. Similarly, our two-sample MR analysis showed that PA does not lead to an increased prevalence of knee OA. In addition, according to our GWAS data source. In terms of PA intensity, our study population engaged in moderate-to-vigorous PA^14^. Therefore, our study can provide support that moderate-to-vigorous PA is not a risk factor for knee OA. We considered that PA did not lead to an increase in the prevalence of knee OA due to body mass index (BMI). It is now well established that obesity is an independent risk factor for the development of knee OA^6^. The effect of obesity on knee OA is not well understood, and the general mechanism is that obesity may affect the metabolic function of cells by altering the regulation of glucose metabolism. In addition, obesity alters mediators of oxidative stress and proinflammatory cytokines. Cytologic alterations lead to histologic cartilage damage and ultimately to the development of knee OA^24^. PA can effectively control BMI (especially moderate-to-vigorous PA)^25^, and since BMI is effectively controlled, obesity as an independent factor will no longer cause knee OA. Notably, although PA also has the potential to cause knee injury, knee injury is also a risk factor for knee OA^5^. Injury prevention programs can effectively reduce the possibility of knee injury^26^. For those concerned about knee OA caused by PA, effective injury prevention programs warrant attention.

## Limitations

Our research has the following shortcomings: (1) The GWAS data we used were taken entirely from individuals of European ancestry, indicating that our results are only applicable to people with European ancestry; further research is needed to prove whether these results are also applicable to other populations. (2) There are various forms of PA, and because of the data sources, we can only analyze PA in general terms and cannot further refine the effects of the various types of PA on knee OA. (3) Because the PA GWAS data were pooled, the conditions for inclusion in the sample varied among institutions. As a result, we also did not have a way to conduct subgroup analyses of elderly people with high BMI or excessive PA. Subgroup analyses that cannot be refined can similarly lead to bias. (4) Our data only apply to moderate-to-vigorous PA, and further exploration is still needed for other intensities of PA.

## Conclusion

In summary, we analyzed the GWAS data of PA and knee OA by using two-sample MR analysis. There was no obvious causal relationship between PA and knee OA. Therefore, PA is not a risk factor for the development of knee OA.

### Supplementary Information

Supplementary Table 1.

## Data Availability

The data that support the findings of this study are available from the corresponding author upon reasonable request.

## Abbreviations

MR: Mendelian randomization
IVs: Instrumental variables
SNPs: Single-nucleotide polymorphisms
GWAS: Genome-wide association studies
PA: Physical activity
OA: Osteoarthritis
IVW: Inverse variance weighted
ORs: Odds ratios
Cis: Confidence intervals
BMI: Body mass index
