# Supplementary material for: Investigating the causal relationship between physical activity and incident knee osteoarthritis: a two-sample Mendelian randomization study
Source: Sci Rep. 2024 Jan 18;14:1663. doi: 10.1038/s41598-024-52175-4 (PMC10796638; doi:10.1038/s41598-024-52175-4)
Supplement: Supplementary file 1 — Supplementary Table 1. [file 41598_2024_52175_MOESM1_ESM.pdf]

Investigating the causal relationship between physical activity and incident knee osteoarthritis: A two-sample Mendelian randomization study

Liufang Huang[1] Yuling Zhang[1] Qian Li[1]
Affiliations:
[1] Department of Rehabilitation Medicine, People's Hospital of Guanghan City

Table 1. The included SPNs

SPNs
rs10821758
rs1160545
rs1265762
rs13201721
rs144949097
rs1625595
rs1691471
rs370935521
rs385301
rs4352559
rs4656648
rs4865512
rs568546
rs7920171
rs8094118
rs9420
rs9903845

SPNs: single nucleotide polymorphism
